# No man is an island: spatial clustering and access to primary care as possible targets for the development of new community mental health approaches

**DOI:** 10.1186/s12913-020-05190-w

**Published:** 2020-04-22

**Authors:** M. Nascimento, B. Lourenço, I. Coelho, J. Aguiar, M. Lázaro, M. Silva, C. Pereira, I. Neves-Caldas, F. Gomes, S. Garcia, S. Nascimento, G. Pereira, V. Nogueira, P. Costa, A. Nobre

**Affiliations:** Centro Hospitalar Psiquiátrico de Lisboa, Avenida do Brasil 53, Lisbon, Portugal

**Keywords:** Join count statistics, Clusters, Primary care, Community psychiatry, Access to healthcare

## Abstract

**Background:**

to understand if patients seen at Centro Hospitalar Psiquiátrico de Lisboa (CHPL) live in geographical clusters or randomly throughout the city, as well as determine their access to the psychiatric hospital and primary care facilities (PCF).

**Methods:**

spatial autocorrelation statistics were performed (*queen* criterion of contiguity), regarding all patients observed at CHPL in 2017 (at the census subsection level), and considering not only their overall number but also main diagnosis, and admission to the psychiatric ward - voluntary or compulsory.

Distance to the hospital and to the closest PCF was measured (for each patient and the variables cited above), and the mean values were compared.

Finally, the total number of patients around each PCF was counted, considering specified radius sizes of 656 and 1000 m.

**Results:**

All 5161 patients (509 psychiatric admissions) were geolocated, and statistical significance regarding patient clustering was found for the total number (p-0.0001) and specific group of disorders, namely *Schizophrenia and related disorders* (p-0.007) and *depressive disorders* (p-0.0002).

Patients who were admitted in a psychiatric ward live farther away from the hospital (p-0.002), with the compulsory admissions (versus voluntary ones) living even farther (p-0.004).

Furthermore, defining a radius of 1000 m for each PCF allowed the identification of two PCF with more than 1000 patients, and two others with more than 800.

**Conclusions:**

as patients seem to live in geographical clusters (and considering PCFs with the highest number of them), possible locations for the development of programs regarding mental health treatment and prevention can now be identified.

## Background

### Social determinants in mental health

Technical literature highlights the importance of various social, economic, and physical environments in which people live, operating at different stages of life and shaping mental health and mental disorders. Social inequalities [1], ethnic density [2] and accessibility to mental health care [3] are some of the social determinants that have been studied during the last years. Physical distance has long been identified as a potential barrier to mental health services, particularly in rural areas [4]. Also, previous studies in other urban cities have demonstrated that, in certain areas, patients appear to reside non-randomly [5] and there are, although sometimes weak, correlations with various ecological factors, such as social organization / safety [6], increased urbanicity [7–9], or increased need of social support [10–15].

### Spatial autocorrelation statistics serving community psychiatry

The development of mathematical and statistical models applied to spatial coordinates enables the assessment of various social, economic and ecological factors that may operate in the balance between mental health and mental disorders. Characterization and localization of the patient’s residence allows not only to identify clusters, but also to cross-link them with the existing primary and secondary care network, in order to understand their relationship with accessibility to care [5, 16].

### Primary care facilities and community psychiatry in central and eastern Lisbon

The Portuguese National Health Service (Serviço Nacional de Saúde - SNS), is a complex and constantly evolving system of care. It provides care for all stages of life, not only regarding illness treatment but also in health promotion and primary prevention. It is organized as a network for a better use of resources.

Primary Care Facilities (PCF) are distributed throughout the country at the community level and constitute the first point of contact with the SNS. The PCF are SNS’s fundamental pillar, where general and public health physicians, nurses, social workers, psychologists and nutritionists provide care to the population. These teams develop interventions regarding health promotion, early disease detection, initiating appropriate treatments or referring to other specialties if necessary. In the case of mental illness, general practitioners are prepared to treat milder forms of common mental disorders, or when necessary, to ask for consultation or referring to Psychiatry. Psychiatric care provides a more specialised care, taking advantage of different services aiming for secondary, tertiary and quaternary prevention [2].

Mental health care in Portugal has been reorganized since the 60s, following international guidelines in an effort to integrate psychiatric care in general hospitals. Although a long way has already been travelled, there are still some psychiatric hospitals operating in Portugal, namely Centro Hospital Psiquiátrico de Lisboa (CHPL) in Lisbon.

According to the 2011 census, CHPL is responsible for providing direct care to the people living in the central area of Lisbon, with a total of 261,350 citizens distributed in a 32.94 km^2^ fully urban area with a population density of 7934 inhabitants/km^2^. It is a predominant urban environment, with heterogenous social and cultural characteristics [17].

In Lisbon, CHPL provides specialized and integrated psychiatric care, framed as an assertive program, using in- and outpatient care, day hospital, occupational therapy, in-home healthcare visits and literacy groups. It articulates with the 15 PCFs operating in this area, based mainly on a referral model, but also on a collaborative approach.

There is a need to establish a closer relationship with the PCF in order to achieve an inspired public health approach that favors a more comprehensive perspective. The ambition to develop and further structure CHPL’s community intervention in Lisbon is lined up with the 2020’s National Mental Health Program [18, 19], which stresses out the need to improve mental health and psychiatric care in the PCF, as well as the development of literacy programs.

To the authors´ knowledge, there are no published papers regarding Lisbon’s reality, focusing on the individual characteristics and localization of the patients that evaluate the environment around them and how it can affect mental illness. This study aims to answer the following questions:

1. Do patients live randomly around the city?
2. Do patients with more severe diagnosis have better access to the healthcare facilities?
3. Are there PCFs with higher prevalence of psychiatric patients?

The authors claim that this evaluation will allow a clearer definition of possible areas of action and the development of tailored community interventions in mental health.

## Methods

### Design and context

The authors carried out a retrospective, observational and analytical study, regarding all patients seen by a psychiatrist at CHPL in the year 2017 and living in Lisbon (*N* = 5161). Data were collected regarding main psychiatric diagnosis (according to the International Classification of Diseases, 10th edition – ICD-10), patients’ need for psychiatric hospitalization that year, and whether that admission was voluntary or not.

Then, based on the 2011’s national public census data, spatial autocorrelation analysis was performed, regarding the existence of possible geographic clusters, considering general number of patients, main diagnosis, need for psychiatric hospitalization and need for compulsive hospitalization (versus voluntary).

The authors also identified the public primary care facilities (PCF, *N* = 15) in the central area of Lisbon, analyzing the number of patients seen in Psychiatry within multiple radius of distance from each PCF, and how many of them had an admission at an acute psychiatric ward (also within a certain distance radius). It is considered that public PCFs are the main focus of the general treatment of all patients, in coordination with all other medical specialties. Therefore, PCFs can be important focal points for the development of initiatives regarding disease prevention, psychoeducation and patient management.

### Participants of the study

Patients seen in Psychiatry at CHPL in 2017 (aged 18 or older), living in the central and eastern regions of Lisbon (according to the Census 2011 data), both in outpatient and inpatient care. In Portugal, the admission into an acute psychiatric ward is considered a last resort to the treatment of patients, therefore it is a measurement of severity of their disorder. Finally, the compulsory admission at an acute psychiatric ward is determined by the court of law, designated for someone who, due to the psychiatric disorders, creates a dangerous situation for legal assets of a significant value, whether personal or external, of a personal or patrimonial nature, and refuses to submit to the necessary medical treatment; and/or does not present the necessary insight to assess the meaning and scope of the consent, when the absence of treatment will severely deteriorate his condition [20].

Individuals without psychiatric diagnosis, or those whose registration did not allow a proper data collection and subsequent statistical analysis were excluded from the study.

### Data collection

Prior to data collection, the study was submitted to and approved by the hospital’s ethics and scientific-pedagogical committees (Hospital deliberations: CCP 02/2019 e CES 02/2019), with particular attention paid to the current General Regulation on Data Protection [21].

The collection of the psychiatric follow-up variables (diagnosis, psychiatric admission in 2017, and compulsory admissions that year) was performed through a direct request of the anonym data to the competent entities of the Hospital, thus avoiding patients’ identification by the authors.

Regarding geographical data, only one of the main authors had access to the patients’ postal code (and only to this information), in order to identify the sub-section of Lisbon (according to the 2011’s census) [17], where they reside. The identification of the census subsection (not the specific address, but the lowest denominator of the census data, which includes a number of buildings located in a small area) allowed patients’ anonymity to be maintained.

Finally, all PCFs located in central Lisbon were identified and georeferenced, thus allowing a subsequent analysis regarding the accessibility of patients to primary care.

### Measures

Regarding main diagnosis, the patients were grouped into the main categories listed below (increasing the statistical power of the statistical analysis performed):

- Organic, including symptomatic, mental disorders (F00–09);
- Mental and behavioral disorders due to use of alcohol (F10);
- Mental and behavioral disorders due to psychoactive substance use, excluding alcohol (F11–19);
- Schizophrenia, schizotypal and delusional disorders (F20–29);
- Manic Episode and Bipolar Disorder (F30–31);
- Depressive episode, recurrent depressive disorder and persistent mood disorders (F32–34);
- Anxiety disorders (F40–41);
- Obsessive-compulsive disorder (F42);
- Reaction to severe stress, and adjustment disorders (F43);
- Dissociative [conversion], Somatoform and Other neurotic disorders (F44–49);
- Eating Disorders (F50);
- Sexual dysfunction, not caused by organic disorder or disease and Gender identity disorders (F52 + F64);
- Disorders of adult personality and behavior (F60–69);
- Mental retardation (F70–79);
- Disorders of psychological development (F80–90);
- Hyperkinetic Disorders (F90);
- Behavioral and emotional disorders with onset usually occurring in childhood and adolescence, (excluding Hyperkinetic disorders) (F91–98);
- Diseases of the nervous system (G00–99).

All patient variables were measured as categorical ones (admissions in 2017, as well as if it was voluntary or compulsory, were measured as yes/no questions). The exact number of patients living around each PCF was considered (continuous variable).

### Data analytic approach

Statistical analysis was carried out using the statistical software R studio©, version 3.4.2, as well as QGIS, version 3.0.2, software applications.

Patients were initially spatially merged according to Census 2011 data, at the census subsection tract. Spatial autocorrelation was measured using joint counting statistics, based on the number of existing pairs of adjacent patient/patient locations (*queen* criterion of contiguity, which includes all units that share a common vertex with each square), and considering 25 × 25 meters to represent the area of each patient (average area of the Census’ subsections in the area of the city here considered). Statistical analyzes were performed for the overall number of patients, main group of disorders, need for acute psychiatric admission, and whether that admission was voluntary or not, considering 95% confidence intervals.

Distance between each patient and each PCF was initially estimated (average of 656 m), as well as distance to CHPL (using “*distances*” and “*multipoint.vars*” functions and packages in RStudio). Average distance to the PCF and to CHPL were compared among patients with a psychiatric admission in 2017 versus patients without admissions in that year, and between compulsory admissions versus voluntary ones (using Student’s t-test). Then, to access proximity and concentration of patients to PCF, the number of patients living within a defined radius was calculated for each PCF - two different radiuses were calculated: 656 and 1000 m, the latter considering that it was the distance needed to include 75% of the total number of patients. Comparative analysis of the data collected, identifying the PCF that had the smallest average distance from the patients’ residency, and the ones that have the biggest number of patients in each radius, was performed. Statistical analyzes were performed for the overall number of patients, main group of disorders, need for acute psychiatric admission, and compulsory admissions. Considering that the present study had a focus on patients’ accessibility to PCF, as they present possible places for development of measures regarding disease prevention and psychoeducation, and also considering that many psychiatric patients have worsened access to PCF (not having an assigned general practitioner), it was accepted that many patients were considered twice, in case their place of residency was associated with more than one PCF.

## Results

In 2017, 5161 patients were treated (1.97% of the corresponding population of the city), with 509 of these patients (9.86%) being admitted to the acute psychiatric ward. Finally, 35.56% of these admissions were compulsory (181 patients). As shown in Table 1, Depressive disorders are the most frequent diagnoses in this population, while Schizophrenia, schizotypal and delusional disorders are the most frequent concerning admissions (voluntary and compulsory). Bipolar disorders represent the third most frequent disorder diagnosed.

**Table 1:** number of patients seen in Psychiatry, and respective acute admissions and compulsory ones – total and by group of diagnosis

Group of diagnoses	N	Total acute admissions in 2017	Compulsory acute admissions in 2017 (%total admissions)
F00–09	290	33	10 (30.3%)
F10	307	69	4 (5.8%)
F11–19	93	34	13 (38.2%)
F20–29	777	107	54 (50.5%)
F30–31	486	88	48 (54.5%)
F32–34	1460	66	11 (16.7%)
F40–41	426	1	0 (0%)
F42	87	2	1 (50%)
F43	189	7	0 (0%)
F44–49	59	3	3 (100%)
F50	38	2	0 (0%)
F52 + F64	57	0	0 (0%)
F60–69	178	12	3 (25%)
F70–79	220	19	6 (31.6%)
F80–90	8	0	0 (0%)
F90	21	0	0 (0%)
F91–98	11	3	0 (0%)
G00–99	36	1	1 (100%)
No definite diagnosis	159	2	2 (100%)
Total	5161	**509**	**181 (35.6%)**

**Fig. 1:**
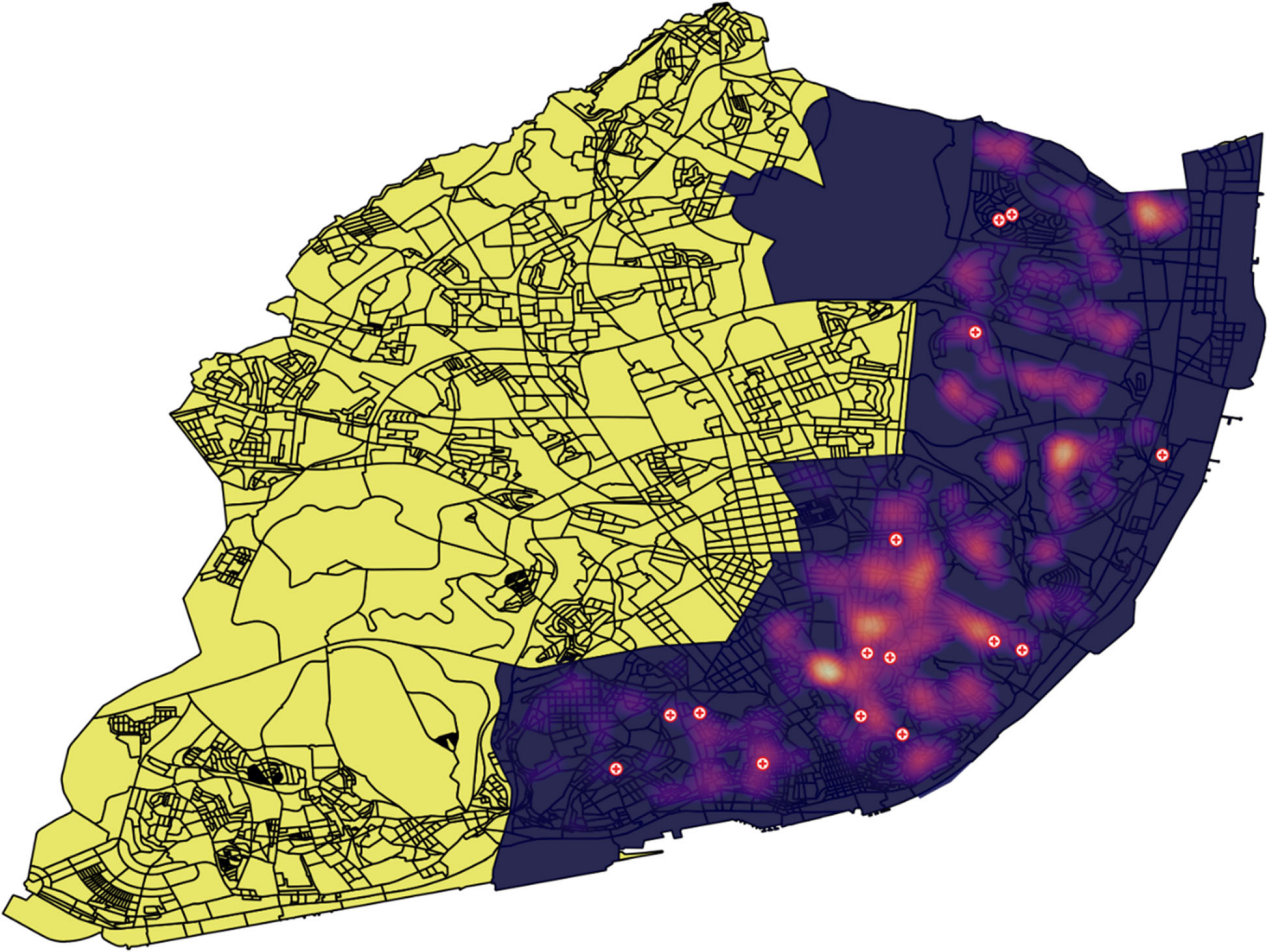
heatmap of all patients living in central and eastern Lisbon. Colored area represents the geographical boroughs assigned to CHPL. Crosses on the map are PCF. Due to data protection, no individual representation of the patients is pictured, but its heatmap

Table 2 shows the *p*-values for the joint count statistics performed, not only for each set of disorders (statistical significance was found in Schizophrenia, schizotypal and delusional disorders and so in depressive and persistent mood disorders, with anxiety disorders also showing a *p*-value of 0.01755), but also for the total number of patients, total number of admissions and compulsory admissions, with the overall number of patients reaching statistical significance. Figure 1 shows a heatmap of all patients living in the central and eastern area, with all the PFC also represented.

**Table 2:** join count statistics p-value regarding all patients followed in 2017

Group of diagnoses	Join count statistics p-value
F00–09	0.6455
F10	0.168
F11–19	0.5125
F20–29	**0.007**
F30–31	0.785
F32–34	**0.0002**
F40–41	0.01755
F42	0.511
F43	0.563
F44–49	0.504
F50	0.5015
F52 + F64	0.5075
F60–69	0.562
F70–79	0.0815
F80–90	0.4995
F90	0.501
F91–98	0.500
G00–99	0.5035
Total patients	**0.0001**
Total admissions	0.1605
Compulsory admissions	0.5705

For the overall number of patients, the average distance to the closest PCF is 0.6564 km, and the average distance to CHPL is 3.6867 km. Table 3 compares these average distances between patients with and without admissions in 2017, as well as compulsory admissions versus voluntary ones. Statistical significance was found in both groups, when comparing average distances to the hospital and not to the closest PCF.

**Table 3:** average distances to the closest PCF and CHPL

		Average distance (km)	
		Closest PCF	CHPL
Acute admissions in 2017	Yes	0.6569	3.8137
	No	0.6564	3.6725
	p-value	0.822	**0.002**
Type of admissions in 2017	Compulsory	0.6576	3.9851
	Voluntary	0.6564	3.7216
	p-value	0.795	**0.004**

Finally, the number of patients living in both 656- and 1000-m radius, sorted by each PCF, is listed on Tables 4 (sorted by total patients, total admissions and compulsory admissions) and 5 (sorted by group of diagnoses).

**Table 4:** number of patients living around each PCF

	Total Patients		Total Admissions		Compulsory admissions	
	656 m	1000 m	656 m	1000 m	656 m	1000 m
UCSP Lapa	157	423	20	45	9	20
UCSP Marvila	110	283	10	20	4	8
UCSP Mónicas	383	697	48	76	21	28
UCSP Olivais	135	444	11	37	3	8
UCSP Penha de Franca	581	1145	68	133	20	51
USF Arco	201	498	22	57	10	24
USF Loios	224	523	15	41	3	9
USF Monte Pedral	323	716	33	70	7	25
USF Oriente	252	516	30	50	5	16
USF Ribeira Nova	284	490	31	58	14	27
USF Sétima Colina	552	1175	60	126	21	51
USF Sofia Abecassis	214	484	19	45	9	23
USF. Baixa	363	873	47	109	18	37
USF Fonte Luminosa / UCSP Alameda	421	858	37	72	14	30
USF Jardins da Encarnacão / USF Vasco Gama	121	430	8	37	1	9
Total	4321	9555	459	976	159	366

**Table 5:** number of patients living around each PCF, sorted by group of diagnosis

	ICD-10 Classifications																																			
	F00–09		F10		F11–19		F20–29		F30–31		F32–34		F40–41		F42		F43		F44–49		F50		F52 + F64		F60–69		F70–79		F80–90		F90		F91–98		G00–99	
	656 m	1000 m	656 m	1000 m	656 m	1000 m	656 m	1000 m	656 m	1000 m	656 m	1000 m	656 m	1000 m	656 m	1000 m	656 m	1000 m	656 m	1000 m	656 m	1000 m	656 m	1000 m	656 m	1000 m	656 m	1000 m	656 m	1000 m	656 m	1000 m	656 m	1000 m	656 m	1000 m
UCSP Lapa	8	23	6	20	4	7	25	64	18	48	38	98	13	37	5	9	6	18	0	7	0	3	0	7	6	13	5	11	0	0	5	5	0	1	1	3
UCSP Marvila	9	20	11	22	0	3	9	31	9	22	34	90	7	20	2	6	4	12	2	4	1	1	2	6	0	6	7	14	0	0	0	1	0	0	1	2
UCSP Mónicas	25	46	32	50	12	17	56	109	34	55	98	190	38	66	2	5	16	26	4	8	2	4	3	8	18	30	12	24	2	2	0	1	2	3	1	1
UCSP Olivais	12	29	8	17	3	4	18	61	11	44	46	130	9	41	1	10	2	14	2	5	0	1	1	1	0	19	7	25	0	2	1	2	0	2	0	5
UCSP Penha de Franca	33	66	32	75	13	25	122	217	41	94	151	299	43	89	9	15	15	37	8	9	9	12	7	14	27	38	18	32	1	2	1	3	1	3	2	5
USF Arco	11	35	10	26	2	9	28	75	29	58	42	113	15	43	4	11	8	16	3	8	3	3	3	9	6	15	13	19	0	0	0	1	1	1	5	7
USF Loios	9	18	5	19	3	4	34	85	31	60	68	160	20	43	1	9	7	18	1	6	3	6	0	3	9	26	18	32	1	2	0	2	0	2	2	3
USF Monte Pedral	18	35	22	41	10	23	35	102	25	57	104	217	25	58	4	10	15	30	4	7	1	6	5	10	8	20	12	24	0	2	2	2	1	1	2	4
USF Oriente	13	25	19	34	10	21	28	72	14	41	79	159	21	40	4	7	11	21	4	7	1	4	5	7	5	14	12	20	0	0	1	2	1	1	2	3
USF Ribeira Nova	13	28	18	31	4	13	47	74	33	50	64	118	26	44	5	9	6	11	4	7	0	2	5	8	9	15	17	21	0	0	0	1	1	1	5	7
USF Sétima Colina	38	63	37	73	10	25	113	204	43	99	133	330	44	92	6	15	14	48	7	12	7	12	6	13	26	39	13	32	1	2	1	2	1	3	1	4
USF Sofia Abecassis	12	29	11	26	2	11	35	73	28	53	48	106	15	45	6	11	9	18	5	7	3	4	4	9	4	13	6	19	0	1	1	4	1	1	3	7
USF Baixa	25	60	31	59	9	20	60	135	28	72	97	235	30	75	3	11	12	31	6	9	1	9	6	9	13	43	12	26	0	3	0	2	3	3	0	4
USF Fonte Luminosa / UCSP Alameda	30	46	27	54	7	13	72	156	48	91	93	224	37	65	7	14	16	36	6	7	5	9	3	7	10	23	13	25	0	0	4	6	0	1	2	2
USF Jardins da Encarnacão / USF Vasco Gama	8	29	6	17	3	4	20	61	9	39	37	130	13	39	1	7	1	14	1	5	0	2	0	1	2	19	6	24	0	1	1	2	0	2	0	5
Total	264	552	275	564	92	199	702	1519	401	883	1132	2599	356	797	60	149	142	350	57	108	36	78	50	112	143	333	171	348	5	17	17	36	12	25	27	62

## Discussion

### Do patients live randomly around the city?

Join count statistics allowed the identification of clusters of patients throughout the city, regarding the overall number of patients and some psychiatric diagnosis. The majority of patients that had contact with the hospital in 2017 does not live randomly in the city and there are areas with a higher density of patients (*p*-value of 0.0001). The same conclusion can be drawn regarding some specific diagnosis, such as depressive disorders, as well as the ones in the schizophrenia spectrum.

The study design doesn’t allow any conclusions on why this happens. It would be interesting to understand if there is any relationship between the diagnosis and the area of the city where the patient lives, as it is recognized the importance of social determinants in the genesis and prognosis of mental disease (and as is was studied in other contexts [7, 10, 13]). Empirically, the authors acknowledge also that there are some neighborhoods in the city where the patients in need of social support are most easily allocated. Further studies are needed to explore this complex and interesting relationship.

Statistical significance was not observed in what could be considered patients with a more severe disorder – namely patients with admissions at the acute psychiatric ward. While there are areas with a higher density of psychiatric patients, it does not seem to lead to the existence of specific locations that could be considered as focal points to more severe cases (*p*-value of 0.1605 for overall admissions and 0.5705 for compulsory ones).

Despite the limitations on extending the conclusions on this topic, it is important to highlight that these patients live in fact in these areas of the city, and any mental health community interventions should take that into account.

### Do patients with more severe diagnosis live closer to healthcare facilities?

In Portugal primary care facilities are considered the first access to healthcare for every citizen. Therefore, it is expected that the average distance to PCFs is smaller than to the hospital, being the general practitioners the first physicians to have contact with these patients.

Patients with a psychiatric admission in 2017 do not seem to live farther away from PCFs, compared to the ones without any admission, suggesting that geographical distance and access to healthcare do not seem to be factors related to disease severity.

However, the same does not apply to the hospital. Patients with at least one admission in 2017 live farther away from it (*p*-value: 0.002) and the same applies to the compulsory admitted patients compared to the voluntary ones (*p*-value: 0.004). It can be considered that, for this population, distance and geographical access to the psychiatric hospital may have a role regarding mental health care and treatment. Therefore, the authors consider that future programs regarding relapse prevention, psychoeducation and mental healthcare should be developed preferably at a community level (namely at PCFs), therefore surpassing this geographical barrier and possibly achieving better outcomes. While there are other studies that regard distance to healthcare as factor towards patients’ treatment [12, 14], this paper expands these results by adding and comparing the PCFs to the psychiatric specialized services.

### Are there PCFs with higher prevalence of psychiatric patients?

Considering a 1000 m radius, there are two PCFs with more than 1000 patients (USF Penha de França and USF Sétima Colina), followed by two others with more than 800 patients (USF Baixa and USF Fonte Luminosa / UCSP Alameda). These PCFs are also consistently the ones with the higher number of patients, when considering each and every group of diagnoses. The authors are aware that the identification of buffers of patients, on a certain radius from each PCF, allows each patient to be part of multiple buffers. Since this study is focused on access to health and community care, it is acceptable for some patients to be counted more than once.

The PCFs with more patients, especially for certain disorders, will become not only the focus for specific strategies regarding their management, but also, they will be targets for better support regarding general practitioners, as promoting specific training to general practitioners is a crucial step in every community program. A better knowledge of the environments’ characteristics, such as recognition of the patients’ “hotspots” and PCF nearby, may allow the restructuring of training contents and interventions accordingly.

### Advantages, limitations, and recommendations

The project becomes relevant for:

1. Studying the geographical organization of psychiatric patients throughout the city, which will allow, in further research, the evaluation of social determinants of the community and their access to primary, secondary and tertiary health care;
2. allowing future improvement of clinical approaches to the psychiatric patient: promotion of local strategies to prevent mental illness and identification of possible places of risk.

Future research will focus on ecological data associated (at the census level) with possible social-economic factors that can be linked to patients with mental disorders.

The authors advocate further development of local strategies with the aid of information disclosed by the present research, and posterior evaluation of their impact.

## Conclusions

Our research suggests that, in this area of Lisbon, patients who contact the hospital seem to present some degree of clustering in terms of their residency, both in general and in specific diagnosis. These findings may help the identification of possible “hotspots” of patients, allowing the development of specific community programs and the discussion of realistic health policies. The promotion of cost-effective initiatives could also prevent readmissions and increase the general quality of life of psychiatric patients.

The identification of the PCF with a higher number of patients can help the further development of tailored measures, namely public health initiatives and psychoeducative programs. If there are PCFs with more patients, especially for certain disorders, not only they can become the focus for specific strategies, but also a target for better support for general practitioners.

Promoting specific training to general practitioners is a crucial step in every community program. A better knowledge of the environments’ characteristics, such as recognition of the patients’ “hotspots” and PCF nearby may allow the restructuring of training contents and interventions accordingly.

The detection of geographic clustering of patients and characterization of PCF psychiatric patients allowed the development of strategic and targeted measures regarding psychiatric care and also the improvement of the relationship between Psychiatrists and General Practitioners. CHPL’s community intervention is now based on a collaborative/consultation approach to provide optimal management of an increasing number of patients with a mental illness within primary care. There are training sessions to general practitioners, prioritizing those with higher prevalence of psychiatric patients. In order to optimize the ways of communication between PCF and Psychiatric settings, an integrated model of communication was stablished through multiple modalities: mobile phone, email or face-to-face, according to the population characteristics and existing needs.

Future research will focus on ecological data associated (at the census level) with possible social-economic factors that can be linked to patients with mental disorders.

## Data Availability

not applicable.

## Abbreviations

CHPL: Centro Hospitalar Psiquiátrico de Lisboa
ICD-10: International Classification of Diseases, 10th edition
PCF: Primary Care Facility
SNS: Serviço Nacional de Saúde
